# PCR and serology confirm the infection of turkey hens and their resilience to histomonosis in mixed flocks following high mortalities in toms

**DOI:** 10.1186/s13071-019-3482-z

**Published:** 2019-05-14

**Authors:** Tarik Sulejmanović, Beatrice Grafl, Ivana Bilić, Barbara Jaskulska, Michael Hess

**Affiliations:** 1 Clinic for Poultry and Fish Medicine, Department for Farm Animals and Veterinary Public Health, University of Veterinary Medicine, Veterinaerplatz 1, 1210 Vienna, Austria; 2 Christian Doppler Laboratory for Innovative Poultry Vaccines (IPOV), University of Veterinary Medicine, Veterinaerplatz 1, 1210 Vienna, Austria

**Keywords:** Turkey, Hen, *Histomonas meleagridis*, Mortality, Histomonosis, ELISA, PCR, Genotyping

## Abstract

**Background:**

Histomonosis, caused by the protozoan parasite *Histomonas meleagridis*, is a severe disease especially in turkeys where it can cause high mortalities. Recently, outbreaks were described in which turkey hens showed no clinical signs despite high mortalities in toms, from which they were separated only by a wire fence. The present study investigated three similar outbreaks of histomonosis whereby in two of them only a few hens were being affected and none in the third. Hens from all flocks were kept until end of production and slaughtered as scheduled. However, in all three cases, the disease progressed in toms reaching nearly 100% within two weeks.

**Methods:**

Following diagnosis of the disease, tissue samples were obtained from toms and hens at necropsy. Environmental dust, cloacal swabs and blood were taken on three successive farm visits within compartments of hens and toms and tested by real-time PCR or ELISA. The DNA from a total of 18 samples positive for *H. meleagridis* was further subjected to conventional PCR utilizing the *18S* rRNA primers and sequenced for phylogenetic analysis.

**Results:**

All tissue samples and some cloacal swabs were tested positive. Dust samples confirmed the presence of *H. meleagridis* DNA that spread within entire houses up to 6 weeks after the first clinical signs of histomonosis. Sequence analysis of the *18S* rRNA locus demonstrated the presence of the same strain in birds of both sexes within each of the turkey houses. Investigation of serum samples two weeks post-initial diagnosis and prior to euthanasia resulted in antibody detection in 73% of toms and 70% of hens. Until the end of the investigation the number of positive hens per farm increased up to 100% with mean OD-values approaching those noticed in toms prior to euthanasia.

**Conclusions:**

For the first time it could be demonstrated that turkey hens kept in the same house as toms became infected during fatal outbreaks in toms. This highlights the value of different diagnostics methods in order to trace the parasite in connection with the host response. The strange phenomenon that only single hens succumb to the diseases despite being infected requires further investigations.

## Background

Histomonosis, caused by the protozoan parasite *Histomonas meleagridis*, is a severe disease especially in turkeys where it can lead to high mortalities [1]. The number of cases increased recently following the ban of efficient chemotherapeutics in Europe and the USA [2]. Although fatality of histomonosis in turkeys is widely reported, mortality can be very low as well [3]. The genetic background of the bird is of minor importance with only slight differences noticed in experimental studies [4]. Similarly, no differences were obtained in experimental studies following infection of male and female turkeys with *in vitro* grown parasites [5, 6].

Different to experimental studies, field reports indicate differences in the outcome between male and female turkeys with males being more susceptible on fattening farms and *vice versa* in breeding stock [7, 8]. It was also reported in the same studies that a simple wire mesh was capable to limit the spread of the disease to a certain compartment within a house. Keeping male and female animals in a single house separated from each other in two different compartments is a widely used management practice in order to gain more space for turkey toms following earlier slaughtering of hens. In such a scenario the spread of histomonosis between two houses was reported but not within the houses where only male birds died which were separated by wire mesh from the hens [8]. Although such reports from the field indicate a different susceptibility of male and female birds, it remains to be elucidated whether female turkeys are infected at all or if they just do not develop the disease despite being infected. In the present study, three mixed turkey flocks were extensively monitored. Clinically, only males died and female turkeys remained unaffected, except for a few dead female birds at the beginning of the outbreak in two of the flocks. In order to determine the infection status of birds, detection of protozoan parasites in environmental dust and cloacal swab samples along with the detection of antihistomonal antibodies by ELISA was performed.

## Methods

### Turkey flocks

Turkey flocks included in the present study were placed on three farms A, B and C, located in different regions of Austria. Each farm consisted of a single house without chicken farms in close vicinity, except for farm C where another turkey farm was located in a distance of about 100 m. Management practices on the farms were identical with supply of day-old birds from the same hatchery and keeping them in the same house until end of rearing. The bedding material consisted of either wood shavings alone or in combination with straw and commercial feed was supplied substituted with coccidiostats (monensin) on all farms. All flocks performed as expected and there were no aberrant incidences reported by the farmers apart from sudden increase in mortalities on farm C upon placement of birds. Each flock consisted of a mixed population of male and female birds, kept in the same house but separated to two compartments by a wire mesh. Flocks were placed at different time points and had no link to each other except that the birds from the 3 flocks originated from the same hatchery. In two of the flocks (farms A and C) hens were placed next to the entrance of the house thus the compartment with male turkeys could only be accessed through the hen compartment. Placement on farm B was *vice versa* with males placed first ones to be accessed when entering the house. Paromomycin was administered to treat turkeys immediately following the diagnosis in all three flocks, with a duration of the treatment from 10 to 14 days and a dose of 12.5 mg/kg b.w. Upon diagnosis, three consecutive visits of affected farms were made to collect clinical data and samples for further analysis. During each visit, 4 dust and 30 cloacal swab samples were collected per compartment for PCR investigation along with 30 blood samples for serology. Liver and caecal tissue samples were taken at necropsy from dead male and female birds that were submitted by the veterinarian in charge upon occurrence of mortalities to diagnose the disease. Apart from just a few dead hens on farms A and B, there were no further mortalities in female birds due to histomonosis on these farms and none at all on farm C. Overall mortalities in hens during the entire cycle of production were 4.75%, 3.7% and 22.5% on farms A, B and C, respectively, with the ones in farm C mainly related to a *Pseudomonas* spp. infection as described below. Detailed information about each of the flocks is as follows: flock on farm A was placed in June 2017 and it consisted of 4470 male and 1990 female turkeys. Within first 4 weeks mortality ranged between 19–29 dead birds/week with a sharp increase in the 5th week of life due to 1847 dead male but only 3 female birds. At 34 days of life, 4 dead birds, 3 males and 1 female, were submitted for necropsy and official diagnosis. All of the necropsied birds had pathognomonic lesions indicative of histomonosis. Just 3 days after, another dead female bird was submitted for necropsy showing typical lesions. First visit of the farm to collect samples took place in the next week, the 6th week of age, when 2460 males had died and *c.*100 of those that remained were quarantined within the female compartment. There were just 4 dead females within this week due to mortalities that were not related to histomonosis but were also not further investigated. The second and third sampling visits took place in August and September 2017, at weeks 9 and 14 of life with only female birds remaining at these sampling points.

The flock on farm B consisted of 3600 male and 2700 female birds which were placed in August 2017. At 26 days of age of the flock, one female bird was submitted for necropsy whereby pathognomonic lesions indicative of histomonosis were observed. There were only 6 fatalities in female and 260 in male birds within the 4th week of age. Mortalities progressed only in males that had to be euthanized by the 6th week of age whereas no further deaths due to histomonosis were recorded in hens until slaughter. The first sample visit took place at 29 days of age of the flock and thereafter in one-week intervals for the second and third farm visit.

Farm C is a farm with a history of repeated outbreaks over the course of 3 years [9]. The affected flock on this farm was placed in November 2017 and it consisted of 3650 toms and 2510 hens. Within the first two weeks 956 toms and 435 hens died due to omphalitis and septicemia related to infection with *Pseudomonas* spp. In the third week only 27 male and 9 female dead birds were recorded with another sharp increase in mortalities in the 4th week of life. On day 20 of life, 17 male birds were found dead after which histomonosis was diagnosed thus the first sample visit took place already on the next day. Three dead toms were obtained for necropsy at 21 days of age after which histomonosis was confirmed and within the next 2 weeks all males had to be culled due to high mortalities. The 2nd and the 3rd farm visit were made in one-week intervals after the first one with no further mortalities noticed in hens due to histomonosis until slaughter.

### Dust samples

**Fig. 1:**
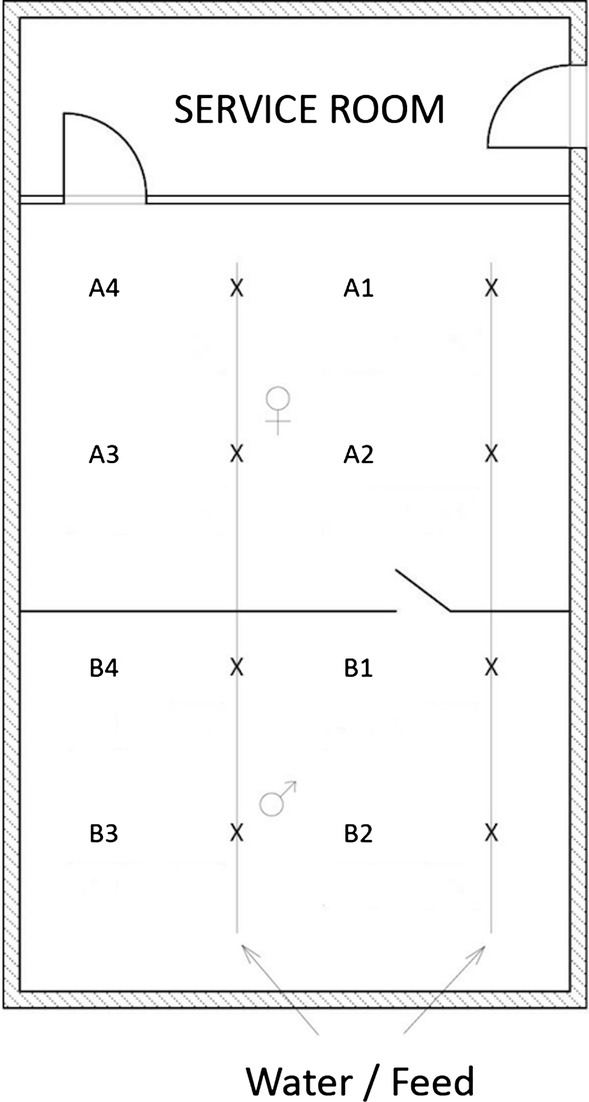
Overview of sampling spots for dust and faecal samples within compartments for turkey toms and hens. A1 to A4 are sampling spots in four corners located in the compartment for hens on farms A and C or the compartment for toms on farm B. B1 to B4 are sampling spots in four corners of the compartment where toms were placed on farms A and C or compartment for hens on farm B

Dust samples were collected separately in 50 ml tubes (Sarstedt, Vienna, Austria) from water and feeding lines in each corner of the two compartments (Fig. 1). Additional dust was sometimes added from nearby walls or air inlets, depending on the availability of the dust on drinking or feeding lines, which differed from house to house or even between visits on the same farm. On farm A, the dust was available for sampling only in the female compartment because male compartment was depopulated at the time of the farm visit for sampling as described in the previous section. A total of 12 dust samples were obtained from this farm. In the other two flocks on farms B and C, dust samples from male compartments were collected at the first and second farm visits whereas the female compartment was sampled during all 3 visits. Thus, the total of 40 samples were collected, 8 from male and 12 from female turkeys on each of the farms B and C (Table 1).

**Table 1:** Overview of mortalities and number of samples taken at farms A, B and C during three consecutive farm visits and at slaughter. Mortalities expressed are only those related to histomonosis

Farm	No. of birds	First farm visit^a^				Second farm visit^a^				Third farm visit^a^		Slaughter		
		Mortality (%)	Dust samples (♂^b^/♀^c^)	Cloacal swabs (♂/♀)	Blood samples (♂/♀)	Mortality (%)	Dust samples (♂/♀)	Cloacal swabs (♂/♀)	Blood samples (♂/♀)	Mortality (%)	Dust samples (♂/♀)	Cloacal swabs (♂/♀)	Blood samples (♂/♀)	Blood samples (♂/♀)
Farm A	4470 ♂ 1990 ♀	95.7 ♂ 0.2 ♀	0/4	30/30	30/30	98.5 ♂ 0 ♀	0/4	0/30	0/30	98.5 ♂ 0 ♀	0/4	30/30	30/30	0/30
Farm B	3600 ♂ 2700 ♀	7.2 ♂ 0.2 ♀	4/4	30/30	30/30	44.9 ♂ 0 ♀	4/4	30/30	30/30	100 ♂ 0 ♀	0/4	30/30	30/30	0/30
Farm C	3650 ♂ 2510 ♀	5.5 ♂ 0 ♀	4/4	30/30	30/30	49.9 ♂ 0 ♀	4/4	0/30	0/30	100 ♂ 0 ♀	0/4	0/30	0/30	0/30

^a^Time elapsed from diagnosis of the disease until each of the three farm visits on farm: A) 7, 28 and 68 days; B) 3, 10 and 17 days; C) 1, 8 and 15 days

^b^Male birds

^c^Female birds

### Cloacal swabs

Cloacal swabs were taken from 30 randomly selected male birds during the first farm visit on farm A, and the first and second farm visits on farms B and C. At the third visit on farms B and C, and at the second and third visit on farm A, sampling of males was not possible due to the fast progression of mortalities and the culling of males in each of the three flocks. In hens, the same samples were obtained from 30 randomly selected birds during three farm visits at each of the farms (Table 1).

### Serum samples

Obtaining the serum samples was done according to the same schedule as for the swab samples. Thirty blood samples were taken from males at each first and second farm visit on farms B and C whereas toms that belonged to farm A were bled only at the first farm visit. Hens from each of the three farms were sampled on three consecutive farm visits. Blood samples were taken concurrently with swab samples and each of the 30 serum samples was paired with a corresponding swab sample from the same bird. Additional 30 blood samples to those paired with swabs were taken from female turkeys in flocks B and C at slaughter (Table 1). After an overnight incubation at 4 °C, all samples were centrifuged at 3300× *g* for 12 minutes (Rotanta 460; Hettich) to separate the serum from clotted blood. All serum samples were tested by the sandwich ELISA according to protocol published by Windisch & Hess [10].

### Faecal samples

In each of the three farms, faecal samples were collected from both male and female turkey compartments on each sampling date and tested for the presence of *Heterakis gallinarum* by the test-tube flotation. The pooled faecal samples within one compartment were collected from the litter below the same spots where the dust was taken and additionally once from the litter in between these spots (Fig. 1).

### DNA extraction

Live histomonads were recruited from caecal tissues obtained at necropsy following cultivation as described earlier [11], without the addition of antibiotics to culture medium. Briefly, approximately 1 g of inflamed caecal tissue and adjacent caecal content were placed in 50 ml tube (Sarstedt) containing 9 ml of Medium 199 supplied with Earle’s salts, L-glutamine, 25 mM HEPES and L-amino acids (GibcoTM, Invitrogen, Vienna, Austria), 11 mg of rice starch (Sigma-Aldrich Handels GmbH, Vienna, Austria) and 15% FCS (GibcoTM, Invitrogen). Histomonad DNA was extracted by the DNeasy Blood and Tissue Kit (Qiagen, Hilden, Germany) following the protocol provided by the manufacturer. The dust samples were subjected to DNA extraction with slight modification of the above mentioned protocol in a way that the equivalent of 0.2 cm^3^ of dust, taken from each sample, was suspended in 300 µl of ATL buffer containing 35 µl of proteinase K (600 mAU/ml) and incubated overnight at 56 °C and 950× *rpm*. Afterwards, crude dust particles were removed by centrifugation at 14000× *rpm* for 1 min and 210 µl of supernatant was used for DNA extraction according to manufacturerʼs protocol. Cloacal swab samples were soaked in 300 μl nuclease-free water and 200 μl of suspension was taken for DNA extraction, which was undertaken with the QIAamp cador Pathogen Kit (Qiagen, Hilden, Germany) according to the manufacturerʼs instructions.

### PCRs and sequence analysis

Extracted DNA samples of cultured ceaca, dust and cloacal swabs obtained from flocks A, B and C were tested with a recently developed real-time PCR based on *18S* rRNA gene [12].

The DNA from a total of 18 samples positive for *H. meleagridis* by real-time PCR, including organ samples, cloacal swabs and dust from both male and female birds on each farm was further subjected to conventional PCR utilizing the *18S* rRNA primers [13]. Amplification products (25 µl) were electrophoresed in a 1.5% Tris acetate-EDTA-agarose gel, stained with ethidium bromide and visualized under UV light (Biorad Universal Hood II, Bio-Rad Laboratories, California, USA). Fragment sizes were determined with reference to a 100 bp ladder (Invitrogen, Life Technologies, Austria). PCR products of the expected size were excised from the gel and purified using the QIAquick Gel Extraction Kit® (Qiagen, Vienna, Austria) according to the manufacturer’s instructions. Direct fluorescence-based sequencing was performed by LGC Genomics GmbH (Berlin, Germany) using the PCR primers. Assembly of sequences and nucleotide sequence alignment were performed with Accelrys Gene, version 2.5 (Accelrys, San Diego, CA). Primer binding sites were excluded from sequences used in analysis. Phylogenetic analysis was performed with MegAlign module of the Lasergene (DNASTAR Inc.) software package applying default settings.

## Results

### Detection of histomonad DNA in cloacal swabs and dust samples

All of the organ samples from three flocks were tested positive by the real-time PCR. For the dust, all except one sample obtained from the compartment of hens in flock B during the first visit at the spot B1 (Fig. 1) were also tested positive (Table 2). This confirmed the spread of *H. meleagridis* DNA within entire houses within 6 weeks after the first clinical signs of histomonosis. Contrary to dust samples, only a limited number of swabs were positive by the real-time PCR except for 26/30 tested swabs obtained from toms at the first farm visit at farm C. A further 13 swab samples from toms were detected positive at the second visit, whereas one positive sample was detected in hens at each of the second and third farm visits on the same farm. On farm A, 3 swabs collected from toms during the first farm visit were positive, without further positive swabs in this flock at any time point. Fourteen swabs obtained from toms at the first visit and one swab obtained from a hen at the second farm visit were tested positive on farm B (Table 2).

**Table 2:** Number of positive/examined samples per farm visit and at slaughter with mean OD values of antibodies in male and female turkeys on farms A, B and C

	Farm	Sex of birds	Dust	Swabs	ELISA	Mean OD values (cut-off = 0.36)
First farm visit	A	♂^a^	×^c^	3/30	21/30	0.95
		♀^b^	4/4	0/30	17/30	0.54
	B	♂	4/4	14/30	10/30	0.42
		♀	4/4	0/30	1/30	0.2
	C	♂	×	26/30	2/30	0.19
		♀	4/4	0/30	1/30	0.22
Second farm visit	A	♂	×	×	×	na
		♀	4/4	0/30	30/30	0.61
	B	♂	4/4	0/30	12/30	0.53
		♀	3/4	1/30	4/30	0.28
	C	♂	4/4	13/30	15/30	0.48
		♀	4/4	1/30	4/30	0.26
Third farm visit	A	♂	×	×	×	na
		♀	4/4	0/30	30/30	0.89
	B	♂	×	×	×	na
		♀	4/4	0/30	14/30	0.46
	C	♂	×	×	×	na
		♀	4/4	1/30	6/30	0.34
Slaughter	A	♂	×	×	×	na
		♀	×	×	×	na
	B	♂	×	×	×	na
		♀	×	×	29/30	1.46
	C	♂	×	×	×	na
		♀	×	×	26/30	1.34

^a^Male birds

^b^Female birds

^c^Birds not available for sampling due to high mortalities/culling

*Abbreviations:* na, not applicable; OD, optical density

### Sequence analysis

**Fig. 2:**
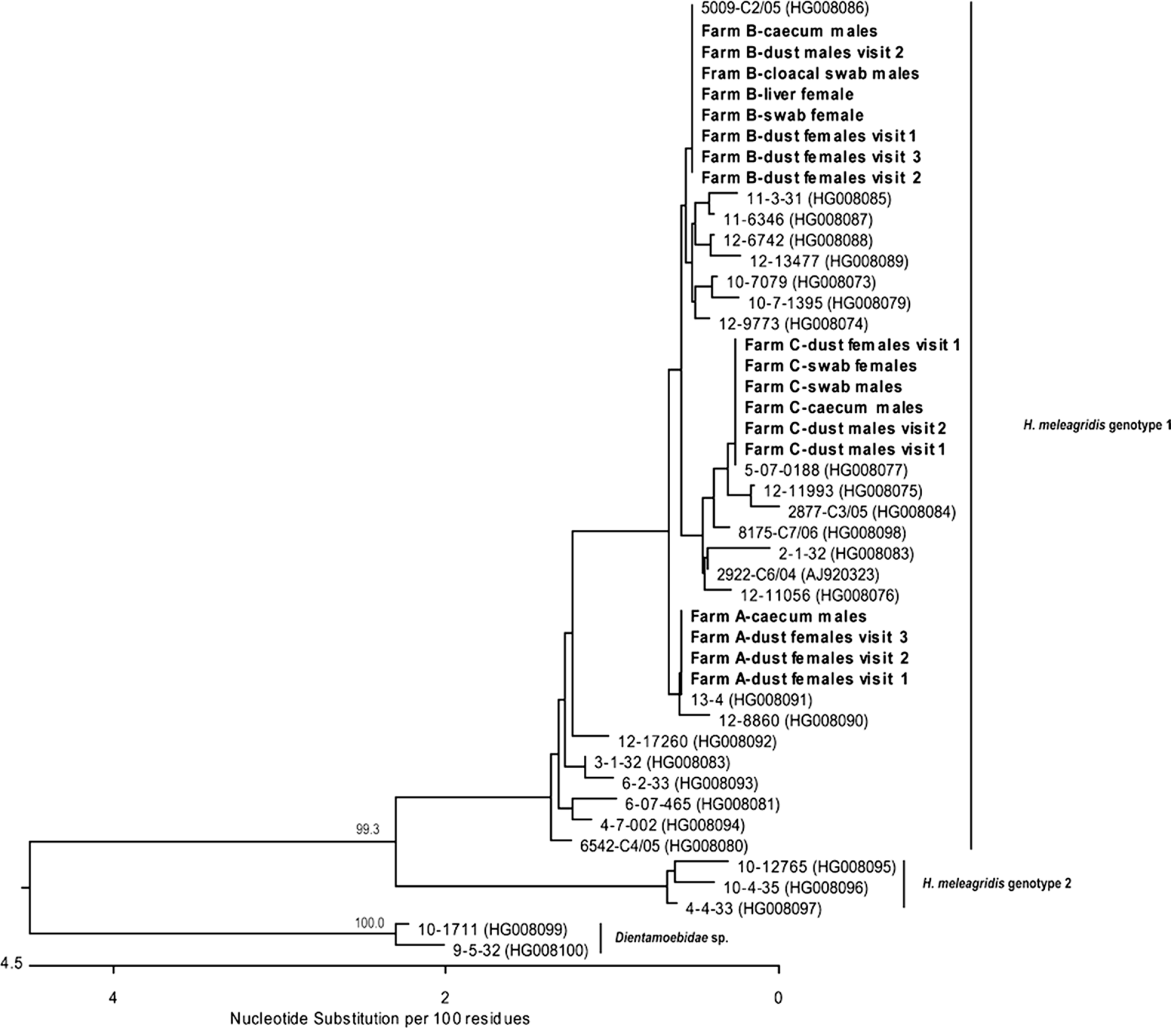
Phylogenetic tree of *H. meleagridis* positive samples based on partial *18S* rRNA locus (*c.*544 bp). Forty-one sequences were used for the analysis. The phylogenetic analysis was performed by the neighbor-joining method implemented in the MegAlign module of Lasergene software package (DNASTAR Inc.) applying default settings. Sequences (*n* = 13) originating from the present study are labelled in bold. Accession numbers for sequences that do not originate from the present study are given in parentheses. The length of each pair of branches represents the distance between sequence pairs. The scale-bar indicates the number of substitution events. Bootstrap values > 70% are given as percentages and are indicated at major nodes

Sequencing of histomonad DNA was performed on 18 samples collected at farms A, B and C. For each farm, sequence data of samples originating from both females and males were obtained. *Histomonas meleagridis* strains involved in all outbreaks clustered within genotype 1, but strains infecting each farm differed from each another (Fig. 2). However, identical nucleotide sequences obtained from samples within the same farm were obtained (Fig. 2), indicating that a single *H. meleagridis* strain was responsible for the infection on the farm independent of the sex of birds.

### Detection of antibodies against *H. meleagridis*

**Fig. 3:**
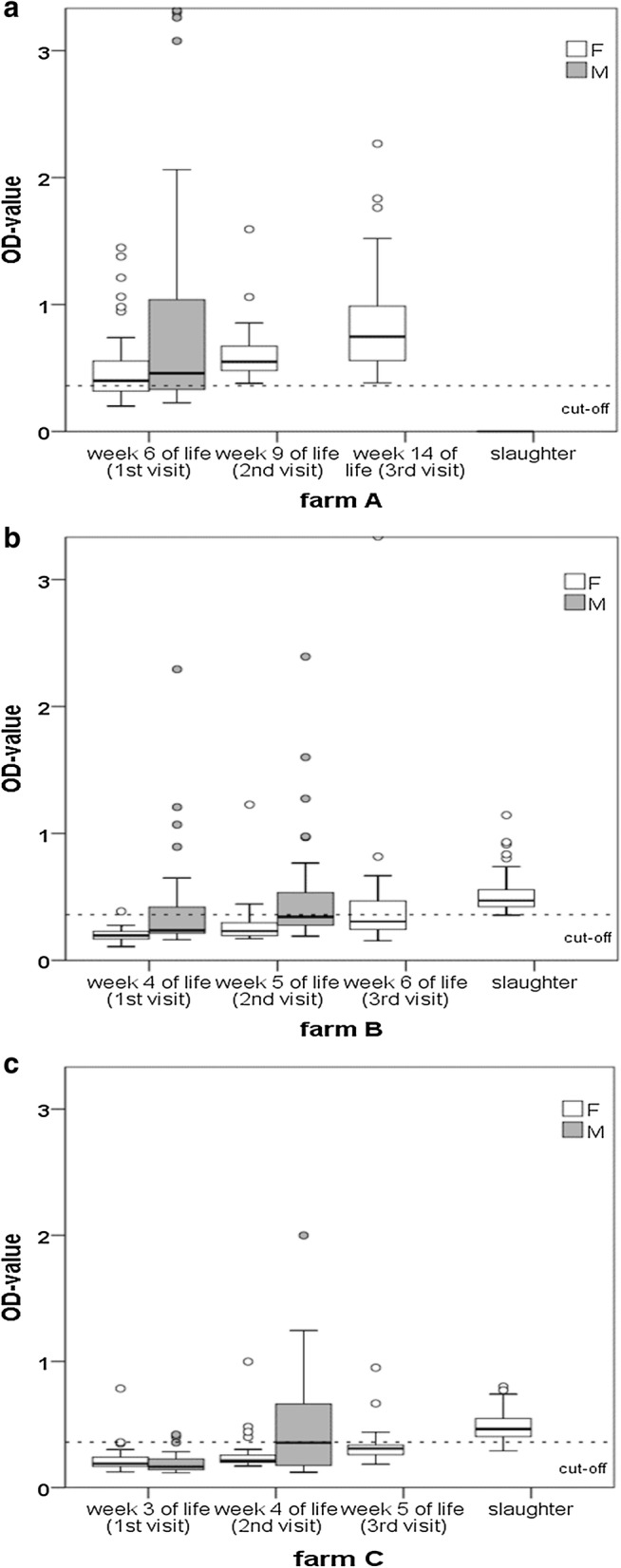
Box plot presentation of optical density (OD) values for IgG antibodies in sera obtained from male (M, grey boxes) and female (F, white boxes) turkeys upon diagnosing of the disease at three sample time points on farms A (**a**), B (**b**) and C (**c**). On farms B and C 60 and 120 samples of sera have been obtained from male and female turkeys, respectively, whereas on farm A 30 and 90 samples from male and female turkeys, respectively, were examined

A total of 21, 10 and 2 out of 30 serum samples from male turkeys of farms A, B and C were positive by ELISA at the first farm visit, respectively (Fig. 3a). At the second farm visit 12 and 15 samples were positive in farm B and C, respectively (Fig. 3b and c). Overall, mean flock titers in male birds on all farms were above the cut-off prior to culling.

In female turkeys, antibodies against *H. meleagridis* were detected in 21, 30 and 30 out of 30 tested samples at the three consecutive samplings from farm A (Fig. 3a). Mean flock titers of hens were above the cut-off value from the first farm visit onwards on this farm. In female birds from farm B antibodies were found in 1, 4, 13 and 29 out of 30 tested samples at the three consecutive farm visit and at slaughter, respectively (Fig. 3b). Mean antibody titers above the cut-off were detected at the third farm visit and at slaughter. In farm C 1, 4, 6 and 26 birds were positive by the *Histomonas* sandwich ELISA at the different farm visits and at slaughter, respectively, although mean antibody titers above the cut-off were only detected at slaughter (Fig. 3c).

### Parasitological examination of faecal samples

Except for a very low number of *Eimeria* spp. oocysts, no nematode eggs were observed at any time point in any of the tested fecal samples.

## Discussion

Due to the imposed ban on effective medication used for prevention or treatment of histomonosis in the EU and the USA [2], an increased number of histomonosis outbreaks have been noticed with varying mortalities, affecting turkey flocks of different size, age and sex, well documented from outbreaks in France [3]. For 2016, 101 outbreaks were reported in the USA which was nearly doubled to those of the preceding year [14].

Interestingly, two reports describe high mortality in male birds whereas female turkeys remain nearly unaffected, despite being raised in the same house albeit in different compartments [7, 8]. Such an observation reflects the situation in other farms raising birds of different sexes simply separated by wire mesh [9]. However, in none of those studies detailed investigations were performed and the questions whether clinically healthy females became infected at all remained unanswered.

The present report describes three outbreaks with a similar outcome of very few or no mortalities in hens kept in the same house with toms who suffered from very severe histomonosis. There is a general consent that both sexes are equally affected based upon the fact that individual sexes are placed in different houses in the field. Contrary to this, a higher infection rate in male birds placed in mixed flocks and loosely separated from females was reported [8, 15], the phenomenon within the focus of the present study. Furthermore, field outbreaks with severe consequences for birds in only one compartment were being noticed in mixed-sex flocks, with peak mortalities occurring in one of the sexes without preferences to a particular sex [3]. Such findings are even more puzzling as experimental data demonstrated that both sexes showed equal susceptibility [5, 6]. However, in none of the aforementioned studies investigations were performed on the infection status of clinically unaffected birds, mainly females, which is somewhat crucial to confirm or exclude the spread of the pathogen. Based on extensive laboratory investigations, we have demonstrated for the first time that the pathogen spreads throughout the entire turkey house, infecting both sexes of birds, irrespective of the presence of barriers. Previously it was suggested that the spread of infection between groups of birds might be interrupted by cage barriers [16]. This was also noticed in field conditions where the disease could be confined within one compartment without further spread but unaffected birds were not investigated [3, 7, 17].

The spread of the pathogen within turkey houses on each of the three farms in the present study is corroborated with the finding of histomonad DNA in a high number of dust samples in both compartments, although the potential is yet to be fully explored [9, 18]. An earlier finding of a rather swift and efficient transmission within the flock after the pathogen is introduced into the turkey house, as demonstrated experimentally, might explain the footprints of the pathogen in environmental samples [19, 20].

The fast spread of the disease among toms with additional infection of hens was also supported by the presence of antibodies determined by ELISA. The *H. meleagridis* ELISA is very specific as antibodies against related flagellates could not be detected [21]. Based upon serology it can be concluded that the infection in all 3 farms started in males as antibodies sharply increase in comparison to females. The start of the disease in toms was also noticed in a recent field report [9]. Overall, antibodies increased with the age of hens and the first positive birds were already noticed 2 weeks after onset of clinical signs. The rapid development is in agreement with data achieved in an experimental study [10]. The rising levels of antibodies in hens until slaughter, as seen in case of farms B and C, indicates a continuous spread and efficient replication of the parasite without causing the disease in these birds. The question remains why only male birds succumb to the disease whereas females remained clinically unaffected, beside a few initial mortalities on two out of three farms with a continuous spread of the infection. Although some swabs positive by PCR indicate a latent infection of hens, isolation of live parasites might be needed for further confirmation.

A few other factors influencing survival of hens in present cases might also be taken in consideration. Due to previously mentioned ban on effective antihistomonal compounds, field veterinarians dealing with severe outbreaks in some countries within the EU have recently started using the aminoglycoside antibiotic paromomycin for metaphylactic treatment of affected flocks with varying results as recently noticed in Austria [9]. As paromomycin was supplied to birds soon after the outbreak of the disease it can be assumed that medication limited further spread. However, the fact that females do not succumb to disease in an identical scenario without usage of the drug points towards other influences, which needs to be resolved in future studies [7, 8]. Finally, an effect of medication might also be questioned due to the fact that no effect was noticed in male birds. A certain sex-related differences in immune response following infection with bacterial or viral pathogens have been noticed in broiler chickens whereby female birds had an earlier humoral response with higher peak antibody titers [22]. Conversely, humoral response of male turkeys was more pronounced in comparison to hens when birds were fed with different diets [23]. Moreover, it is known that sex hormones play role in modulating the immune responses [24]. Considering the dependence of *H. meleagridis* on the presence of certain bacteria it can easily be hypothesized that the microbiome of the birdʼs gut plays a significant role in establishing an infection [25]. Once again the sex of the birds was found to be of importance as it has a large effect on intestinal microbiota [26]. However, this needs to be further elaborated in context of histomonosis.

The sequence analysis of histomonads involved in the present cases demonstrated the identity of parasites within each of the affected farms, thus excluding the involvement of different strains infecting male and female turkeys. In general, there are two genotypes of *H. meleagridis* based on the *18S* rRNA gene, genotype 1 and genotype 2, with the latter occurring less frequently [13]. Only slight differences in pathogenicity of different clonal isolates within genotype 1 have recently been observed in an experimental study [27]. In comparison, Tyzzer [28] observed variations in virulence of naturally occurring histomonad strains. However, Lund et al. [29] concluded that histomonosis often appears as a mixed infection with different *H. meleagridis* strains of varying virulence, a feature which could influence the outcome of the disease.

## Conclusions

Histomonosis is a parasitic disease of gallinaceous birds with the ability to induce severe mortalities in commercial turkeys raised for meat production. In the present report, a difference in disease progression was observed between male and female turkeys housed together in three separate field outbreaks. Applying suitable diagnostic methods, we have demonstrated, for the first time, that the female turkeys become infected without substantial clinical outcome. Consequently, a recently developed real-time PCR in combination with serology were proven to be adequate techniques to elucidate the infection status of hens. Since similar scenarios are recently being more often observed in the field, this phenomenon should be closely monitored in order to provide an insight into the epidemiology of *H. meleagridis* and the underlying mechanisms of histomonosis.

## Data Availability

The datasets used and/or analyzed during the present study are presented in the article and its additional files. Representative sequences were submitted to the GenBank database under the accession numbers: HG008091 (Farm A), HG008086 (Farm B) and HG008077 (Farm C).
